# Identification and expression analysis of starch branching enzymes involved in starch synthesis during the development of chestnut (*Castanea mollissima* Blume) cotyledons

**DOI:** 10.1371/journal.pone.0177792

**Published:** 2017-05-23

**Authors:** Liangke Chen, Dan Lu, Teng Wang, Zhi Li, Yanyan Zhao, Yichen Jiang, Qing Zhang, Qingqin Cao, Kefeng Fang, Yu Xing, Ling Qin

**Affiliations:** 1 College of Plant Science and Technology, Beijing Collaborative Innovation Center for Eco-Environmental Improvement with Forestry and Fruit Trees, Beijing University of Agriculture, Beijing, China; 2 Beijing Key Laboratory for Agricultural Application and New Technique, Beijing University of Agriculture, Beijing, China; 3 Key Laboratory of Urban Agriculture (North China), Ministry of Agriculture, Beijing, China; 4 College of Landscape Architecture, Beijing University of Agriculture, Beijing, China; Beijing Forestry University, CHINA

## Abstract

Chinese chestnut (*Castanea mollissima* Blume) is native to China and distributes widely in arid and semi-arid mountain area with barren soil. As a perennial crop, chestnut is an alternative food source and acts as an important commercial nut tree in China. Starch is the major metabolite in nuts, accounting for 46 ~ 64% of the chestnut dry weight. The accumulation of total starch and amylopectin showed a similar increasing trend during the development of nut. Amylopectin contributed up to 76% of the total starch content at 80 days after pollination (DAP). The increase of total starch mainly results from amylopectin synthesis. Among genes associated with starch biosynthesis, *CmSBEs* (starch branching enzyme) showed significant increase during nut development. Two starch branching enzyme isoforms, CmSBE I and CmSBE II, were identified from chestnut cotyledon using zymogram analysis. CmSBE I and CmSBE II showed similar patterns of expression during nut development. The accumulations of CmSBE transcripts and proteins in developing cotyledons were characterized. The expressions of two *CmSBE* genes increased from 64 DAP and reached the highest levels at 77 DAP, and SBE activity reached its peak at 74 DAP. These results suggested that the CmSBE enzymes mainly contributed to amylopectin synthesis and influenced the amylopectin content in the developing cotyledon, which would be beneficial to chestnut germplasm selection and breeding.

## 1. Introduction

The polysaccharide, starch, comprises a major component of many plant storage tissues. Both transitory and storage starch is composed of the linear polymer, amylose, and the branched polymer, amylopectin. Amylose is comprised of α-1, 4-linked glucose (Glc) monomers, whereas amylopectin contains 5% α-1, 6-linked Glc in addition to linear regions of α-1, 4-linked Glc[1–4]. Long-chain amylose has molecular weight of ~10^5−6^, while amylopectin has a much higher molecular weight (~10^7−8^) and many short-chain branches (approximately 15–20 Glc units per chain) [5]. Amylopectin is the major constituent of starch, and represents approximately 70% of maize (*Zea mays*) starch [4] and ≥ 85% of the starch in rice (*Oryza sativa*) [6–7].

Several classes of enzymes are involved in starch biosynthesis, including ADP-glucose pyro-phosphorylase (AGPase), granule-bound starch synthase (GBSS), soluble starch synthase (SSS), starch branching enzyme (SBE), starch debranching enzyme(DBE), and plastidial starch phosphorylase (Pho1), moreover GBSS, SSS, SBE and DBE have multiple isoforms [4, 8–12]. SBE (EC 2.4.1.18), which is a glucosyl transferase that catalyzes the formation of the α-1, 6-linkages of amylopectin, introduces branches in starch chains and thus plays a vital role in amylopectin synthesis. During the branching process, a malto-oligosaccharide chain is removed from a growing glucan polymer by cleavage of an internal α-1, 4 bond, and then transferred to the C-6 position of the same, or an adjacent chain, forming an α-1, 6 branch point on a linear chain [11]. SBE not only catalyzes the formation of new branch, but also adds a new nonreducing end in the starch molecular. Starch synthesis can continue at the new nonreducing end. Therefore, SBE determines the branching pattern in amylopectin, and influences the amount of starch [13]. Most plant species have two subtypes of SBE (SBE I and SBE II), and that SBE II has two isoforms in some species (SBEIIa and SBEIIb). Each of these enzymes has distinct role in the starch synthesis, and SBE I tends to use amylose as substrate and transfers longer glucan chains, while SBE II tends to use amylopectin as substrate and transfers short chains [11, 14–17]. Previous studies had shown that SBE activity correlates with amylopectin biosynthesis in developing endosperm, and the differential expressions of SBE isoforms greatly influence starch content and physicochemical properties [11]. The expressions of genes encoding SBEII isoforms from a range of species were strongly associated with the abundance of starch granules [18], and the alternative splicing of *SBEII* gene in bean (*Phaseolus vulgaris* L.) had been reported that it led to distinct enzymatic properties [19].

Starch is also abundant in chestnut (a perennial fruit tree), where it can account for 46~64% of the nut dry weight [20]. Chestnut is cultivated worldwide, and Chinese chestnut is native species in China [21]. According to Food and Agriculture Organization of the United Nations (FAO) statistics, the harvested area of Chinese chestnut was approximately 297,000 hm^2^ in 2014, accounting for 56% of the harvested area worldwide and contributing to 82% of the world production [22]. Chestnut is mainly consumed after roast and high amylopectin content (approximately 3 fold of amylose) affects cooking quality by making the nut more glutinous [21, 23–24]. Despite the abundance of starch in the chestnut seed, little is known about the mechanistic relationship among starch synthesis-related enzymes, starch granule properties and key genes in starch synthesis pathway.

In this study, we identified the full length sequences of two starch branching enzyme genes, *CmSBE I* and *CmSBE II*, from developing chestnut cotyledons. Their expression patterns and activities in developing cotyledons were evaluated in order to investigate the function of SBE in chestnut starch accumulation. These results provide insights into the molecule mechanism of chestnut starch synthesis and can contribute to germplasm utilization and proceeding quality enhancement.

## 2. Materials and methods

### 2.1 Plant materials

Samples were collected from Chinese chestnut cultivar ‘Yanshanhongli’ with 15-year-old in the Chestnut Experiment Station in the Huairou district of Beijing. Pollination was controlled by removed the male flower and bagging once female flowers were appeared, and then artificial pollination were carried at full-bloom stage to ensure that all the flowers were pollinated by cultivar ‘Huaijiu’ at the same time. The nuts were collected equally from the east, west, south, and north facing parts of each tree at 60, 64, 68, 71, 74, 77, 80, and 83 DAP. The nuts from each tree were viewed as one replicate, and all the samples were three replicates. After removal of the shell and embryo, the cotyledons were frozen in liquid nitrogen and stored at -80°C for further studies.

### 2.2 Protein isolation from developing cotyledons

One gram of developing cotyledons was homogenized and suspended in 2 mL pre-chilled soluble protein extraction buffer (100 mM HEPES buffer [pH 7.4], 2 mM EDTA, 20% glycerol [v/v],8 mM MgCl_2_, 5% β-mercaptoethanol, 1% protease inhibitor cocktail (Sigma USA)). The homogenate was centrifuged at 20,000 g for 10 min at 4°C. The protein concentration of the supernatant was quantified using the Quick Start Bradford Protein Assay Kit (BIO-RAD, USA). The supernatant was then frozen in liquid nitrogen and stored at -80°C until further use.

### 2.3 Non-denaturing polyacrylamide gel electrophoresis (PAGE) and enzyme activity staining

Native-PAGE was conducted at 4°C as previously described [25]. Briefly, 20 μg of protein was applied to each lane of a native polyacrylamide slab-gel. After electrophoresis, the gel was rinsed three times in ddH_2_O, and then was equilibrated with 10 mM MES (20 mL, pH 6.2) three times for 10 min each. The gel was then incubated in 20 mL reaction buffer (50mM MES [pH 6.2], 10mM glucose-1-phosphate, 1mM adenosine monophosphate, and 25 units of rabbit muscle phosphorylase α [Sigma]), at 25°C on a rotary shaker (33 rpm) for 15 h. After incubation in the reaction buffer, white bands formed in the gel. SBE zymograms were stained with a 0.02% I_2_ (w/v), 2% KI (w/v) solution and immediately photographed.

### 2.4 Cloning and sequencing of *CmSBE I* and *CmSBE II* full-length cDNAs

All primers involved in the various cloning steps are listed in S1 Table. A total of 0.5 μg RNA was used for cDNA synthesis in a 50 μL reaction using Reverse Transcriptase M-MLV (Takara, Japan). PCR amplification was performed in a 20 μL volume using ExTaq DNA polymerase (Takara, Japan). PCR conditions were: 94°C for 4 min; 28 cycles at 95°C for 30 s, 53°C for 30 s, and 72°C for 20 s; extension at 72°C for 8 min. RT-PCR conditions: 94°C for 3 min; 39 cycles at 95°C for 30 s, 53°C for 30 s, and 72°C for 20 s; melt curve 65°C to 95°C (BIO-RAD 1000 CFX. USA). *CmSBE I* was amplified from chestnut cotyledon cDNA using the primers SBEI-F and SBE I-R, *CmSBE II* was amplified using the primers SBE II-F and SBE II-R, and 5’ and 3’ rapid amplification of cDNA ends (RACE) experiments were carried out according to the manufacturer’s instructions (Invitrogen, USA). The gene-specific primers (GSP) used in the 3’ RACE were I3'-GSP and II3'-GSP, respectively. Three 5’ gene-specific primers were used to amplify each the *CmSBE I* and *CmSBE II* genes: I5'-GSP1, I5'-GSP2 and I5'-GSP3 for *CmSBE I*, and II5'-GSP1, II5'-GSP2 and II5'-GSP3 for *CmSBE II*. A Gel Extraction kit (AXYGEN, USA) was used to purify the PCR products, each of which was then ligated into the pMD-19T plasmid (Takara, Japan). The constructs were then transformed into *Escherichia coli* (Takara, Japan) and the recombinant plasmids were purified and sequenced.

### 2.5 SDS-PAGE and immunoblotting

To examine the accumulation of SBE proteins in chestnut cotyledons, extracts from the cotyledons containing 20 μg of total protein were loaded into 4–12% Bis-Tris gradient gels and separated by gel electrophoresis at 120 V. The Thermo PageRuler Prestained Protein Ladder (Thermo, USA) was used to estimate the molecular weight of the protein bands. Proteins separated by SDS-PAGE were electroblotted (200 mA for 120 min) onto nitrocellulose membranes (Bio-Rad, USA). The resulting membranes were probed with primary polyclonal antibodies raised against SBE I and SBE II from Chinese chestnut. Anti-CmSBE I was developed using the peptides C-KNKNDEDWSMNE (peptide 1) and C-EEDKVIVFERGD (peptide 2), while anti-CmSBE II was developed using the peptides C-KKKDEDWRMGDI (peptide 1) and C-DDLKSLIDKAHE (peptide 2). Cysteine residues were added to these peptides to enable conjugation to either keyhole limpet protein or ovalbumin, and affinity purified mouse immunoglobulin raised against CmSBE I and CmSBE II was produced (Abmart, China). The membranes were incubated in blocking buffer at 4°C overnight. The anti-sera were diluted 1:1000 with blocking buffer (10 mM Tris-HCl, pH 7.5, 0.5 M NaCl, 3% BSA) and the immunoblots were incubated overnight with shaking at shaker (Qilinbeier, China) at 4°C. The membranes were then rinsed three times with TBST (10 mM Tris-HCl, pH 7.5, 0.5 M NaCl, 0.02% Tween 20) for 10 min. A secondary goat anti-mouse immunoglobulin-alkaline phosphatase conjugated antibody (Sigma, USA) was subsequently used at 1:1000 (diluted with blocking buffer) for 2 h at 37°C with gentle rotation. Finally, the membranes were washed three times with TBST for 10 min. 33 μL 5% 5-bromo-4-chloro-3-indolyl phosphate (BCIP) and 66 μL 5% nitroblue tetrazolium (NBT) (Solarbio life science, China) were diluted into 10 mL working solution (0.1 M NaCl, 0.05 M MgCl_2_, 0.1 M Tris). The membranes were incubated to visualize the immunoreactive bands. The reaction was stopped after 2 minutes by rinsing the membrane with water and the bands were observed using camera (Nikon, Japan).

### 2.6 Mass spectrometry (MS) and conserved SBE domains

MS analyses of tryptic digests of SBE proteins were conducted to verify the immunoblot results, as described by Qin et al [26]. The selected bands corresponding to those that immuno-reacted with the SBE antibody in the Western blot were excised from a coomassie blue stained SDS-PAGE gels for in-gel trypsin digestion. Each gel slice was resuspended in buffer A (2% acetonitrile [ACN], 0.1% formic acid [FA]) and centrifuged at 20,000 g for 15 min. Ten μL supernatant was loaded onto a 2 cm C18 trap column (Thermo scientific, USA) connected to an Ultimate 3000 nano LC (Dionex, USA). The peptides were eluted onto a 10cm analytical C18 column (inner diameter 75 μm) packed in-house. The samples were loaded at 4 μL/min for 5 min, then the 34 min gradient was run at 400 nL/min starting from 8 to 30% buffer B (98% ACN, 0.1% FA), linear gradient for 5 min, from 30% to 60%, followed by a 3 min linear gradient to 80%, and maintenance at 80% for 8 min, before a final return to 5% for 2 min. Peptide fragmentation and detection was performed using a Q Exactive (Thermo scientific, USA) mass spectrometer. The instrument settings were as follows: full MS resolution 70,000, dd-MS2/dd-SIM resolution 17,500, multiply charged (2_ and 3_) ions rising above predefined threshold intensity were automatically selected for MS/MS analysis. Peptides were selected for MS/MS in an operating mode with a normalized collision energy setting of 28%. The applied electrospray voltage was 1.8 kV. Automatic gain control (AGC) was used to prevent overfilling of the Orbitrap: 1×10^4^ ions were accumulated in the ion trap to generate collision-induced dissociation CID spectra. For MS scans, the m/z scan range was 350–2,000 Da. The MS/MS spectral data were searched against the SwissProt database using Data Analysis software (Bruker Daltonics) and the MASCOT in-house search engine (MatrixScience). Sores generated by the MOWSE algorithm were reported as -10log_10_ (*p*), where p is the probability that the observed match is a random event. IONS scores of > 22 were considered significant (p<0.05). Conserved domains were predicted by CD-Search in the NCBI database [27–30].

### 2.7 SBE activity in developing chestnut cotyledons

Proteins were extracted from cotyledons at different time points after pollination, and SBE activity in the extracts was determined as previously described [31].The optical density of the final reaction solution was measured at 660 nm and the reaction without incubation at 37°C for 30 min was used as the negative control. SBE activity was calculated as OD 660(%) = [OD660 (t0)–OD660 (t30)] / OD660 (t0) and a 1% decrease of the starch-iodine-blue intensity was considered to correspond to one SBE activity unit/g fresh weight (FW).

### 2.8 Semi-quantitative PCR and qRT-PCR

Total RNA was extracted from cotyledons at various stages and various tissues (cotyledon, bud, root, leaf) using a Quick RNA Isolation Kit (Huayueyang biotechnology, China) according to the manufacturer’s instructions. A total of 0.5 μg RNA templates were used for cDNA synthesis in a 50 μL reaction using Reverse Transcriptase M-MLV (Takara, Japan). PCR amplification was performed in a 20 μL volume using ExTaq DNA polymerase (Takara, Japan). Semi-quantitative PCR was used to measure the expressions of *CmSBE I* and *CmSBE II* and other genes with a BIO-RAD C1000-Touch Thermal Cycler. The qRT-PCR primers involved in this experiment are listed in S2 Table. A chestnut *β-Actin* gene (GenBank accession number: EV253704) was used as control. PCR conditions were: 94°C for 4 min; 28 cycles at 95°C for 30 s, 53°C for 30 s, and 72°C for 20 s; extension at 72°C for 8 min. RT-PCR conditions: 94°C for 3 min; 39 cycles at 95°C for 30 s, 53°C for 30 s, and 72°C for 20 s; melt curve 65°C to 95°C (BIO-RAD 1000 CFX, USA).

### 2.9 Amylose and amylopectin content

Starch granules were prepared from chestnut cotyledons as previously described [32]. Amylose and amylopectin contents (%, mg/mg dry weight) were determined using the dual wavelength spectrophotometer method [33]. One hundred mL of distilled water was added to 1 g of dried chestnut starch, and a 2.5 mL sample was pipetted into a 100 mL meter glass and 40 mL distilled water was added. Finally, 0.5 mL iodine reagent (2% KI, 0.2% I_2_) was added into the solutions [34]. The pH of the solutions was adjusted to 3.5 with 0.1 M HCl. These solutions were then diluted to 50 mL with distilled water and allowed to stand for 20 min prior to measure absorbance in10 mm quartz cells using a UV spectrophotometer (Metash, China). The average values from three independent samples were calculated.

### 2.10 Scanning electron microscope (SEM) observation of cotyledons

Chestnut cotyledons were fixed immediately after collection as previously described [35]. To examine cross-sections, dried chestnut cotyledons were cut with a razor blade and the surface was sputter coated with gold. After using a fine coater (JEOL JFC-1200) for 120 s, the morphology of the starch granules was examined by SEM (JEOL-5600, Japan) using the secondary electron mode at 15 kV. The granule areas from 3 random fields of view were individually measured using Image J and average values were calculated.

### 2.11 Starch pasting properties

Chestnut starch properties were measured using a Rapid Visco Analyser (RVA) (Perten instruments, Australia). First, 2.52 g starch and 25.48 g distilled water were added to an aluminum RVA canister, giving a total constant sample weight of 28 ± 0.01 g. In all the samples, the moisture level was maintained at 14%. The initial stirring speed was 960 rpm to mix the sample, followed by 160 rpm for the duration of the experiment. The mixture was held at 50°C for 1 min, heated to 95°C in 3.7 min, held at 95°C for 2.5 min, then cooled to 50°C in 3.8 min and a final step at 50°C for 2 min. The total procedure lasted 13 min. The resulting curves were analyzed using TCW3 software (Perten instruments, Australia), and all the tests were carried out in triplicates.

## 3. Results

### 3.1 Amylopectin accounts for most of starch accumulation during cotyledon development

**Fig 1:**
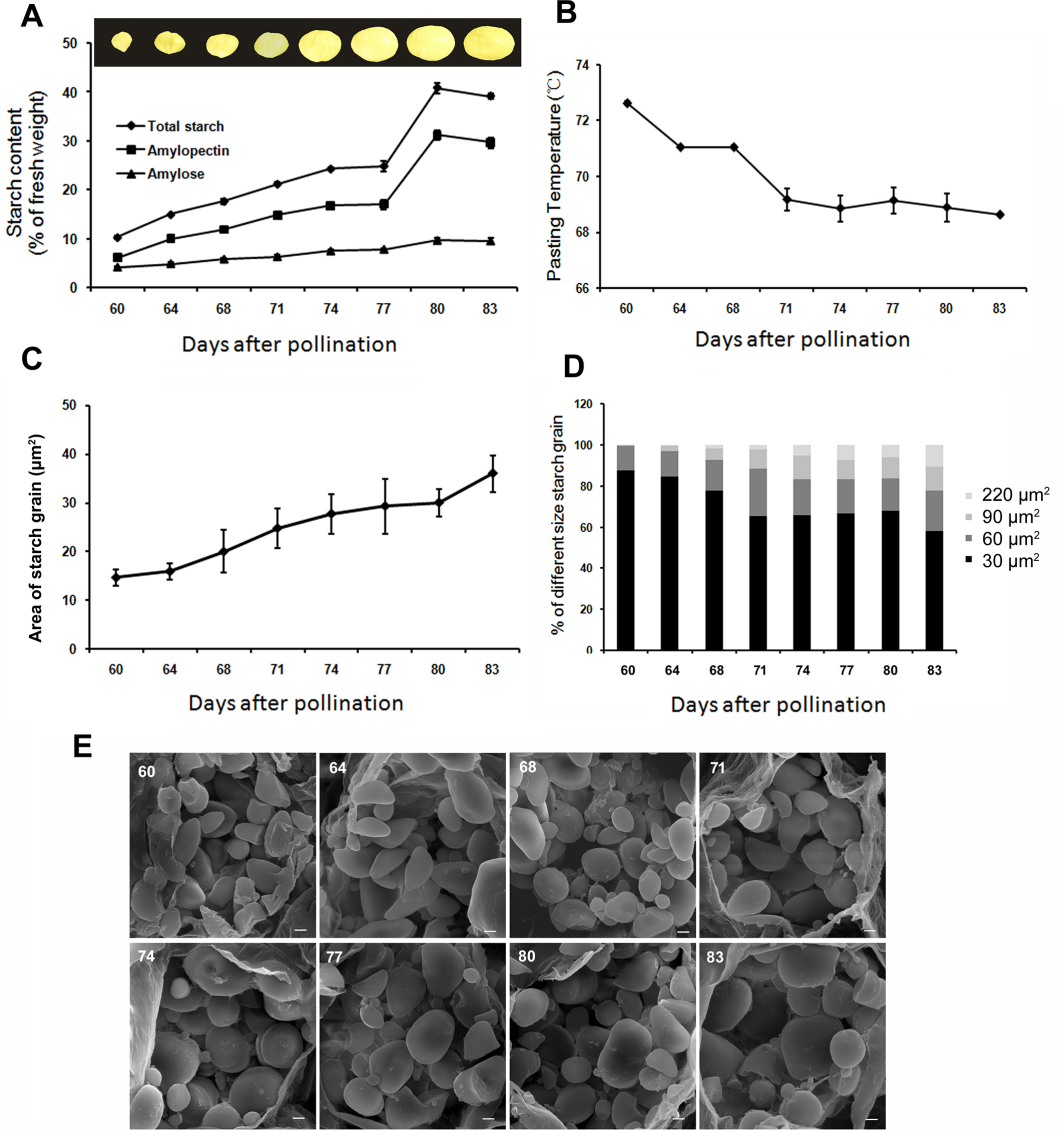
Starch content, pasting temperature and granule size in developing chestnut cotyledons. A. Total starch, amylose and amylopectin contents during chestnut development. B. The pasting temperature of chestnut starch at different time points. C. Average starch granule areas at different developmental stages. D. The proportion of different-size starch granules. E. Scanning electron micrographs of starch granules. The number of 60, 64, 68, 71, 74, 77, 80, and 83 represents days after pollination. Scale bars, 5 μm. Data are means ± SE (n = 3).

Total starch accumulated during development of chestnut cotyledon and reached peak at 80 DAP. Level of amylopectin was 1.5 fold greater than that of amylose at 60 DAP when cotyledons appeared, and increased to 3.2 fold at 80 DAP peak when nuts were mature. The accumulations of amylopectin showed a similar increasing trend and occupied 76% of total starch content at its peak (31% of fresh weight) at 80 DAP. Thus, the increase in total starch content was mainly due to amylopectin synthesis. Amylose levels were low, accounting for approximately 5% of the fresh weight and remained constant throughout development (Fig 1A). Starch collected from the earliest developmental stage of cotyledon had the highest pasting temperature (72.63°C; Fig 1B), and the pasting temperature decreased by 4°C over the studied developmental period, indicating that the starch became easier to gelatinize during cotyledon growth. The average starch granule areas were relatively low at the early stages and increased steadily during development, as did the number of large starch granules (Fig 1C). During the whole development, the proportion of starch granules > 30 μm^2^ increased from 12% to 42% (Fig 1D). Various types were observed when the morphology of the starch granules was visualized using SEM (Fig 1E), such as irregular spheroids or ellipsoids with smooth surfaces, and starch granules grew in size during cotyledon development.

### 3.2 Temporal and spatial expression of *CmSBE I* and *CmSBE II* in chestnut cotyledons

**Fig 2:**
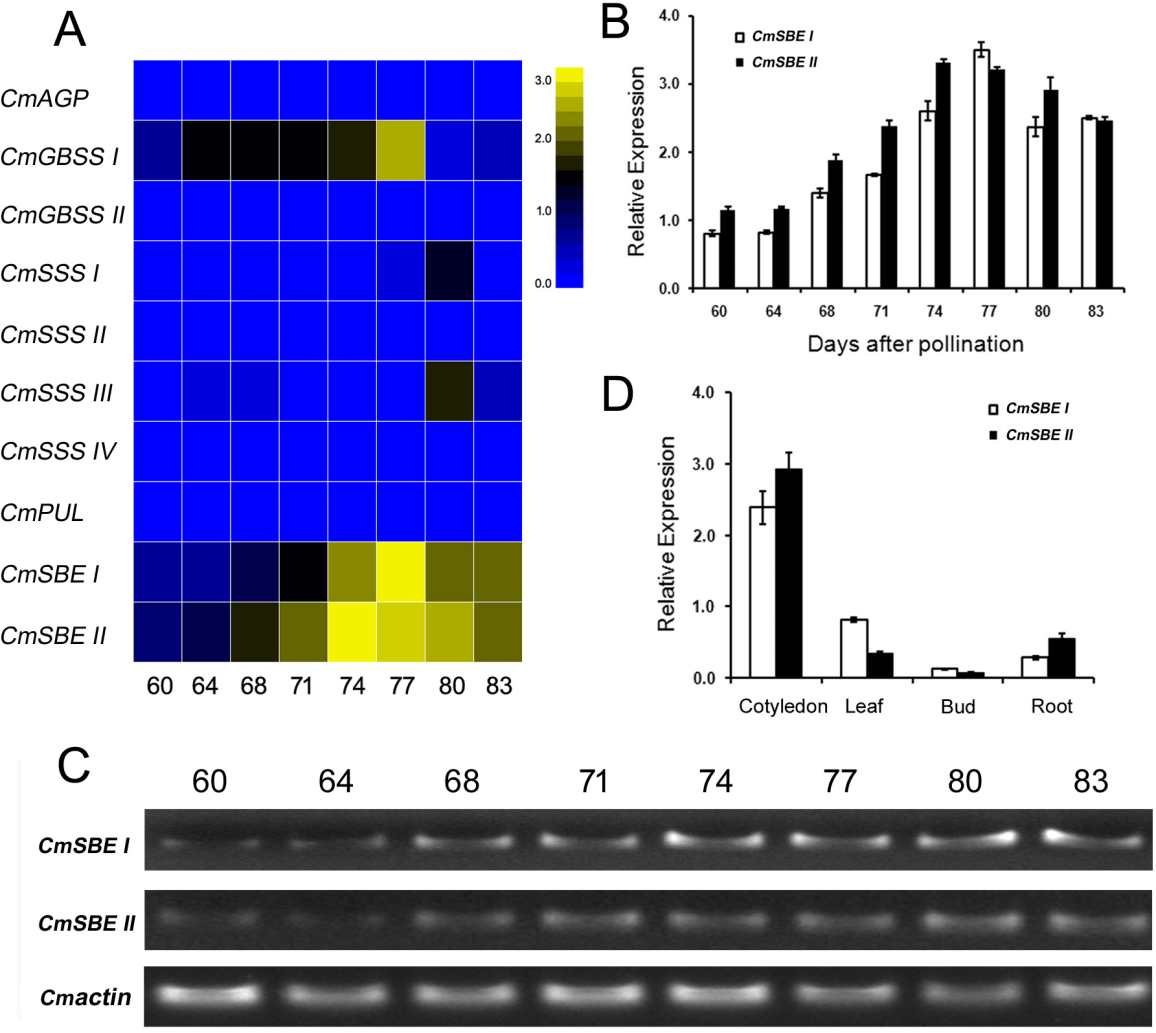
The expression patterns of genes associated with starch synthesis in developing cotyledon, and *CmSBE* expression pattern in different organs. A. Heat map showing gene expression of *AGP*, *GBSS*, *SSS*, *PUL* and *SBE* during chestnut development. B and C. The relative expressions of *CmSBE I* and *CmSBE II* genes in chestnut cotyledon at different days after pollination (DAP) measured by qRT-PCR and semi-quantitative PCR, respectively. The expression of chestnut *β-Actin* was used as a control. The number of 60, 64, 68, 71, 74, 77, 80, and 83 in A, B and C represents days of cotyledon development after pollination. D. The relative expression levels of *CmSBE I* and *CmSBE II* in cotyledon (74 DAP), leaf, bud and root. Values represent the means ± SE (n = 3).

We investigated the expression of genes associated with starch synthesis during cotyledon development. Although the expressions of *CmSSS I*, *CmSSS III* and *CmGBSS I* increased during development, the expressions of *CmSBE I* and *CmSBE II* correlated with amylopectin levels showed the most obvious increase, which was consistent with the role of these genes in starch granule enlargement (Fig 2A, S3 Table). Hence, we choose *CmSBE* as target gene to conduct the following experiment. *CmSBE I* and *CmSBE II* transcript levels were also measured using qRT-PCR and semi-quantitative PCR, and they showed similar patterns, gradually increased in the early stages, and then reached peak at 74 and 77 DAP, respectively. After this peak the expression showed slight decline (Fig 2B, Fig 2C). When the expression was investigated using qRT-PCR in different organ such as cotyledon, leaf, bud and root (Fig 2D), both genes had the highest expression level in cotyledon and the lowest in buds, with a 19.9 fold of *CmSBE I* and 38.4 fold of *CmSBE II* differences in expression between cotyledons and buds, respectively.

### 3.3 Identification of CmSBE isoforms

**Fig 3:**
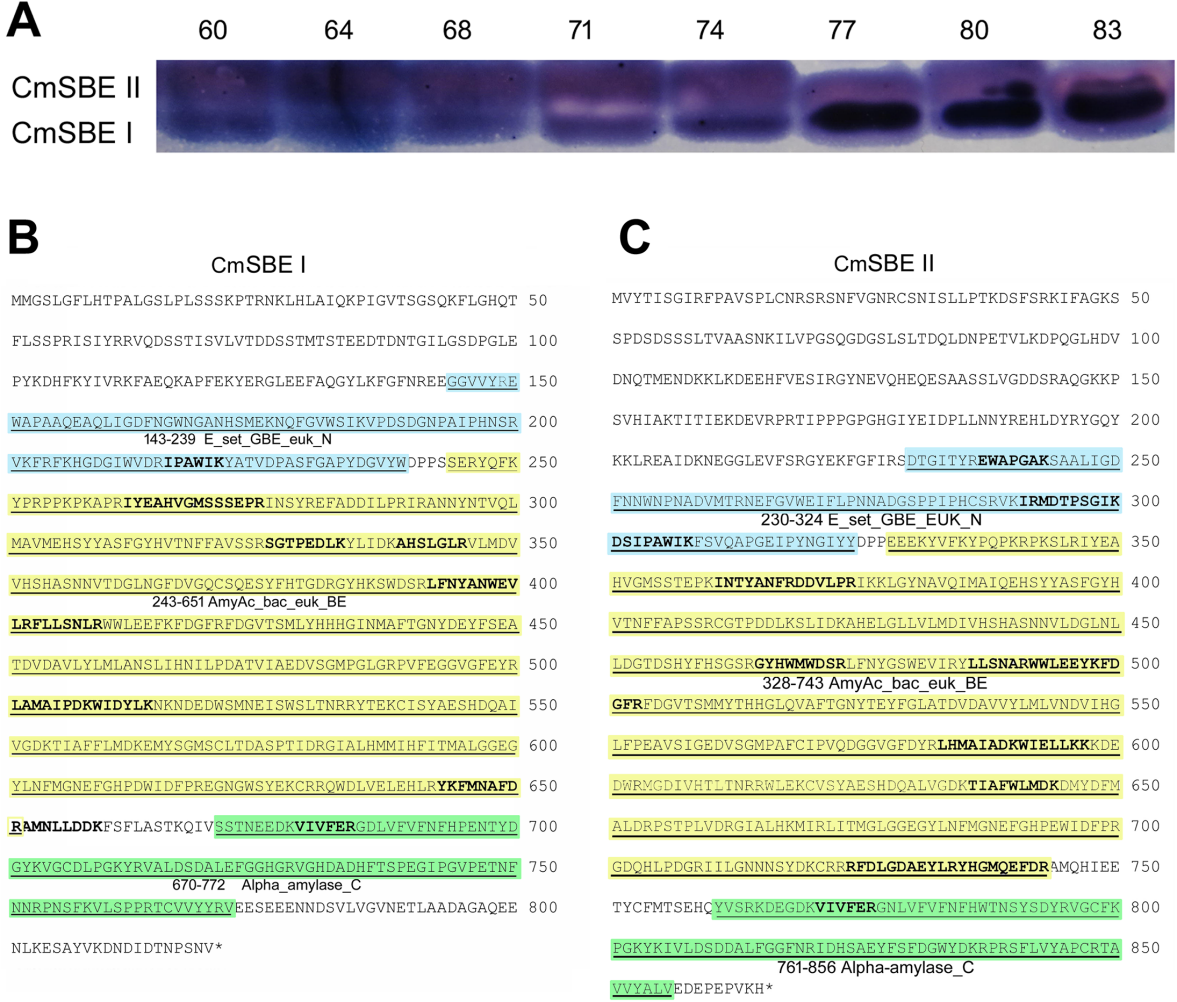
Identification and conserved domain analysis of CmSBE I and CmSBE II. A. The identification of CmSBE I and CmSBE II by native-PAGE. The number of 60, 64, 68, 71, 74, 77, 80, and 83 in A and B represents days of cotyledon development after pollination. B and C. Peptides of CmSBE I and CmSBE II were detected by LC-MS/MS and their conserved domains were predicted by CD-Search tool in the NCBI database. Bold fonts in the sequences indicate peptide fragments detected by LC-MS/MS. Blue regions associated with amino acids 143–239 of CmSBE I (B) and 230–324 of CmSBE II (C) indicate the conserved domain of E_set_GBE_euk_N. Yellow regions associated with amino acids 243–651 CmSBE I (B) and 328–743 of CmSBE II (C) indicate the conserved domain of AmyAc_bac_euk_BE. Green regions associated with amino acids 670–772 of CmSBE I (B) and 761–856 of CmSBE II (C) indicate the conserved domain of Alpha-amylase_C.

To characterize the isoforms of SBE in chestnut, zymogram assay based on staining starch with iodine was performed by native-PAGE (Fig 3A). The gel showed two bands in each sample, corresponding to SBE I and SBE II.

*CmSBE I* and *CmSBE II* were cloned by RACE using cDNA template derived from Chinese chestnut cotyledon. The open reading frame (ORF) of *CmSBE I* was predicted to be 2,460 bp in length (GenBank accession number: KY429029), encoding a putative protein of 820 amino acids with a molecular weight of 92.91 KDa. The full length ORF of *CmSBE II* was predicted to be 2,595 bp in length (GenBank accession number: KY429028), encoding a protein of 865 amino acids (98.50 KDa).

The bands in the zymogram gel corresponding to CmSBE I and CmSBE II were excised (S1 Fig) and MS analyses of tryptic digests of proteins eluted from the gel slices were conducted to identify the constituent proteins and peptides. After searching the SwissProt database and the MASCOT in-house search engine, both CmSBE I and CmSBE II were identified. The peptide sequences covered sequence of 12% and 15% for CmSBE I and CmSBE II, respectively. The detected peptides were shown in bold in Fig 3B and 3C and were listed in S4 Table.

Conserved domains in CmSBE were predicted by CD-Search of the NCBI database (Fig 3B and 3C). SBE I and SBE II have three shared domains. Domain one (blue region) is named E_set_GBE_euk_N (NCBI Acc. cd02854), which corresponds to an N-terminal early set domain associated with the catalytic domain of eukaryotic glycogen branching enzyme (also called 1, 4 alpha glucan branching enzyme). The second domain (yellow region) is AmyAc_bac_euk_BE (NCBI Acc. Cd11321), which is an alpha amylase catalytic domain found in bacterial and eukaryotic branching enzymes. The third domain (green region) is a C-terminal all-beta domain, named Alpha_amylase_C (NCBI Acc.Pfam02806), which presents in proteins belonging to 1, 4-alpha-glucan-branching enzymes.

### 3.4 Protein expression and activity of CmSBE I and CmSBE II in chestnut cotyledons

**Fig 4:**
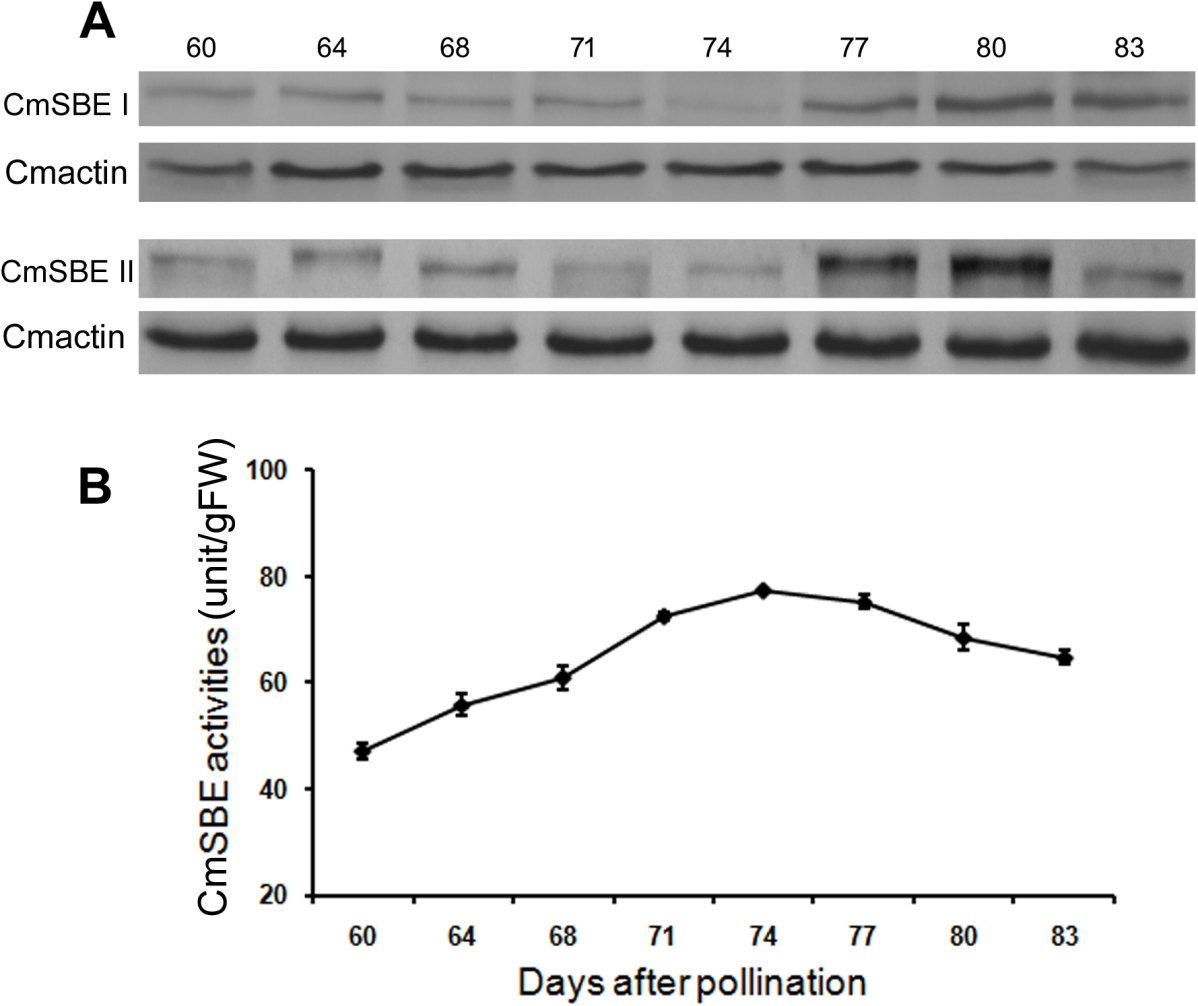
The protein expression patterns and enzyme activity of CmSBE. A. Immunoblot analysis of cotyledons showing the accumulation of CmSBE I, CmSBE II and Actin (control). B. CmSBE activity in chestnut cotyledons in development stages. The number of 60, 64, 68, 71, 74, 77, 80, and 83 represents days of cotyledon development after pollination.

Western blot analyses were performed to detect the expression patterns of CmSBE I and CmSBE II in cotyledon (Fig 4A). Although the relative amounts of CmSBE I and CmSBE II differed, they exhibited the same pattern of accumulation during development with relatively low levels in the initial time points and increased around 77 DAP.

Total CmSBE activity during chestnut cotyledon development was analyzed spectrophotometrically using starch/ iodine staining assay. The activity of CmSBE increased steadily from 60 DAP to 74 DAP, and declined slightly in the latter period of chestnut development (Fig 4B).

## 4. Discussion

Starch is the most abundant component of chestnut, accounting for approximately 46%-64% dry weight [20], but the underlying mechanisms of biosynthesis have not been well characterized. Amylopectin accounts for almost 75% of total starch in ripening chestnut, and the remaining 25% was amylose [21]. In our study, the ratio of amylopectin to amylose increased significantly through chestnut cotyledon development. The patterns of total starch and amylopectin accumulation were similar. Starch with higher amylopectin content tends to be more glutinous and have a waxy quality, for example, waxy corn starch is consisted mostly of amylopectin and has very little amylose [36]. Starch granules in the later developmental stages have a lower ratio of amylose to amylopectin, resulting in less effective packing of the starch chains and making the starch easier to gelatinize [37]. It has also been proposed that the interaction among amylopectin chains can affect the swelling and pasting properties of rice starch [38]. The amount of amylopectin in chestnut starch has also been positively correlated with its waxy and glutinous properties [20]. Roasted chestnuts with high glutinosity are popular among the consumers in China and thus developing chestnut cultivars with higher amylopectin is an important target for breeders.

As an important amylopectin synthesis enzyme, the number of SBE isoform varies among species. Three isoforms of SBE (SBEI, SBEIIa, and SBEIIb) were detected in maize, rice, wheat and barley [25, 39–42], while in *Vigna radiate* [43,44], *Ipomoea batatas* [45]and *Phaseolus vulgaris* [46], SBE had been classified into SBE I and SBE II isoforms. In *Arabidopsis*, SBE I did not expressed and its function was unknown, while SBE II had been classified into SBE 2.1 and SBE 2.2 [47]. Transcriptomic analysis of Chinese chestnut revealed two SBE unigenes; however, the isoforms and sequences of SBE were not defined [21]. In this study, we firstly identified and characterized two chestnut SBE isoforms involved in starch metabolism in chestnut by SDS-PAGE and MS analysis.

SBE gene expression patterns and activities have critical effects on the synthesis of amylopectin, and consequently influence the characteristics of starch granules. Suppression of SBE I, IIa and IIb in barley grains was reported to result in increased levels of amylose, generating starch containing only amylose that was highly resistant to enzymatic digestion [48]. Silencing both *SBE IIa* and *SBE IIb* in wheat increased amylose content from 25.5% to > 70% [49]. Over expression of branching enzyme can result in increased branch numbers of amylopectin in rice transgenic lines [50]. As shown in the heatmap, despite the fact that *GBSS I*, *SSS I* and *SSS III* showed changes during chestnut development, the expressions of *SBE I* and *SBE II* had the most obvious changes compared to other starch synthesis-related genes (Fig 2A). Synthesis of amylopectin requires the coordination of SSS and SBE enzymes, as well as DBE. The interaction of SSSI-SSSIIa, SSSI-SBEIIa, SSSI-SBEIIb, SSSIIa-SBEIIb, and SBEI-SBEIIb is common in wheat, maize, and rice [18]. SBE I and PUL interactions had also been reported in rice [51]. We inferred that SBEs might contribute to amylopectin synthesis in chestnut through coordination with other enzymes, and this would be a key point in future studies.

The content and components of amylopectin contribute to starch properties and then affect the starch processing quality [52]. As a dicotyledon plant, chestnut accumulates starch in cotyledon rather than monocots’ endosperm [53]. It may lead to the different functional mechanism of starch synthesis and accumulation in chestnut compared to maize, rice and barley. Study on chestnut SBE and then to amylopectin will be beneficial to the modification of starch component and the breeding program of chestnut.

## Supporting information

S1 Fig SDS-PAGE gel for LC-MS/MS. Black triangles indicate protein marker bands of 100KDa and 70KDa. The black box indicates the gel slice that was isolated for LC-MS/MS.
(DOCX)

S1 Table List of primers sequences used in the cloning of *CmSBE*. *CmSBE I* was amplified using SBEI-F and SBE I-R primers and *CmSBE II* was amplified using SBEII-F and SBE II-R primers. The gene-specific primer (GSP) used in 3’ RACE were I3'-GSP and II3'-GSP. Both *CmSBE I* and *CmSBE II* gene had 3 gene-specific primers for 5’ RACE. I5'-GSP1, I5'-GSP2 and I5'-GSP3 for *CmSBE I* gene, and II5'-GSP1, II5'-GSP2 and II5'-GSP3 for *CmSBE II* gene.
(DOCX)

S2 Table List of primers sequences used in qRT-PCR of the heat map. (DOCX)

S3 Table Correlation analysis between the gene expressions and some index of starch properties. ** P < 0.01, *p < 0.05; Past tem = pasting temperature, Area = average area of starch content, 30 μm^2^ = the proportion of starch granule ≤ 30 μm^2^, 60 μm^2^ = the proportion of starch granule between 30 μm^2^ and 60 μm^2^; 90 μm^2^ = the proportion of starch granule between 60 μm^2^ and 90 μm^2^; 220 μm^2^ = the proportion of starch granule between 90 μm^2^ and 220 μm^2^.
(DOCX)

S4 Table List of peptides from the SDS-PAGE slice identified by LC-MS/MS. Name, name of chestnut protein matched to the peptide; MS/MS peptide sequence, detected peptide sequence of chestnut by MS/MS; Pep_score, the score of the peptide; Pep_ expect, credibility evalution of the peptide; Mr_exp, expected relative molecular weight of the peptide; Mr_calc, calculated relative molecular weight of the peptide; Identified protein, identified protein using the MASCOT research engine and Swissprot2015 (Taxonomy: Viridiplantae [Green Plants] [35,297 sequences]) Species, the species of the identified protein; NCBI Acc, accession number from NCBI database of identified protein; Protein description, matched protein description; Mascot score, score obtained from MASCOT for each match; SC, amino acid sequence coverage for the identified proteins; Protein coverage was calculated on the basis of the amino acids (aa) identified and matched to the total number of aa in the protein sequence.
(DOCX)
