# Supplementary material for: Identification and expression analysis of starch branching enzymes involved in starch synthesis during the development of chestnut (Castanea mollissima Blume) cotyledons
Source: PLoS One. 2017 May 23;12(5):e0177792. doi: 10.1371/journal.pone.0177792 (PMC5441625; doi:10.1371/journal.pone.0177792)
Supplement: S1 Fig — Black triangles indicate protein marker bands of 100KDa and 70KDa. The black box indicates the gel slice that was isolated for LC-MS/MS. (DOCX) [file pone.0177792.s001.docx]

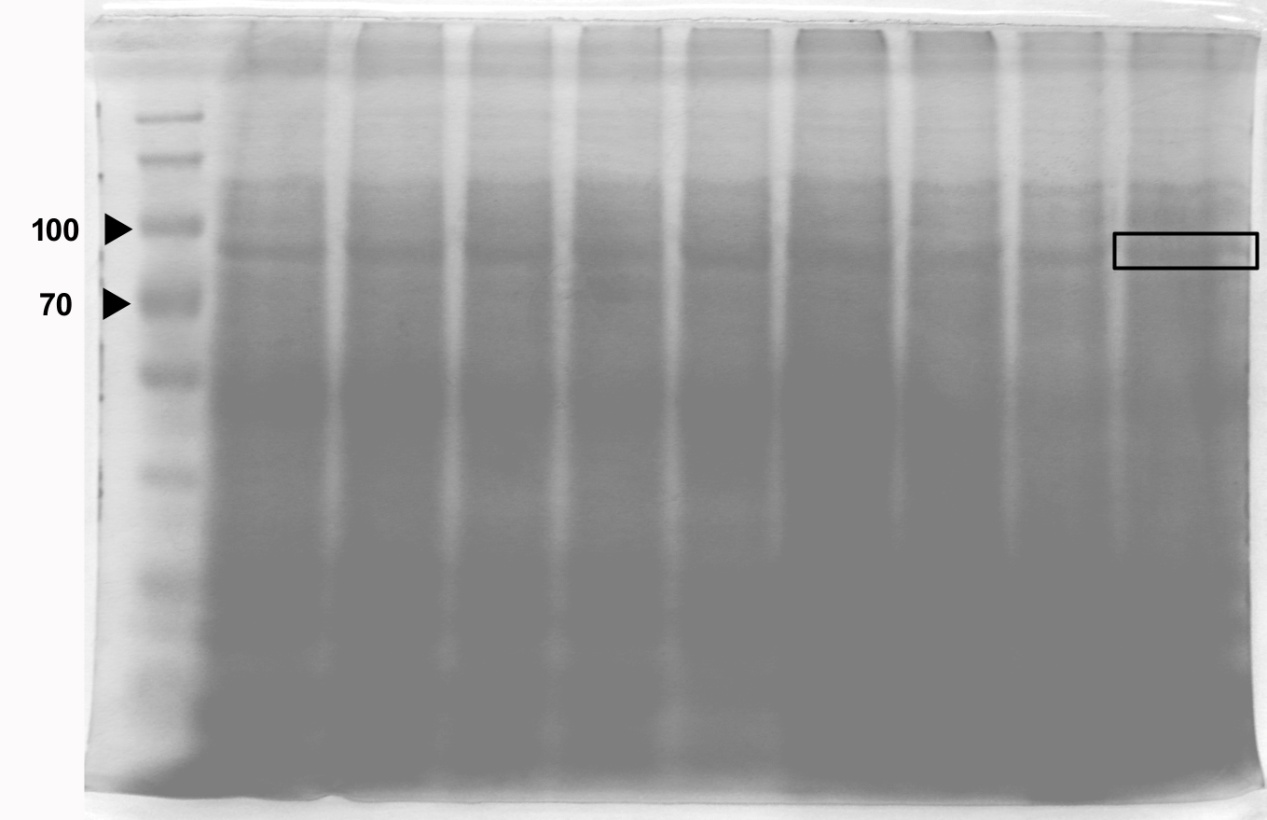


**S1 Fig. SDS-PAGE gel for LC-MS/MS.** Black triangle indicates protein marker bands of 100 KDa and 70 KDa respectively. Black box indicates the gel slice has been sent to be detected by LC-MS/MS.
