# Supplementary material for: Identification and expression analysis of starch branching enzymes involved in starch synthesis during the development of chestnut (Castanea mollissima Blume) cotyledons
Source: PLoS One. 2017 May 23;12(5):e0177792. doi: 10.1371/journal.pone.0177792 (PMC5441625; doi:10.1371/journal.pone.0177792)
Supplement: S1 Table — CmSBE I was amplified using SBEI-F and SBE I-R primers and CmSBE II was amplified using SBEII-F and SBE II-R primers. The gene-specific primer (GSP) used in 3’ RACE were I3'-GSP and II3'-GSP. Both CmSBE I and CmSBE II gene had 3 gene-specific primers for 5’ RACE. I5'-GSP1, I5'-GSP2 and I5'-GSP3 for CmSBE I gene, and II5'-GSP1, II5'-GSP2 and II5'-GSP3 for CmSBE II gene. (DOCX) [file pone.0177792.s002.docx]

**S1 Table. List of primers sequences used in the cloning of *CmSBE*.**

| Primer name | Sequences |
| --- | --- |
| SBEI-F | 5’-ATGATGGGCTCTTTGGGT-3’ |
| SBEI-R | 5’-TTACACATTGCTTGGATTGG-3’ |
| SBEII-F | 5’-ATGGTCTACACCATCTCAGG-3’ |
| SBEII-R | 5’-TCAGTGTTTAACGGGTTCC-3’ |
| I3'-GSP | 5’-CGATACTATCACCGCCTCGCACAT-3’ |
| II3'-GSP | 5’-CGACTCTGATAGATAAAGCCCACG-3’ |
| I5'-GSP1 | 5'-CGACGAATACGAGGCAAA-3' |
| I5'-GSP2 | 5'-CGATTGGAGGACGAGGAT-3' |
| I5'-GSP3 | 5'-CGAGAGGAATGGCTGGA-3' |
| II5'-GSP1 | 5'-CGATCCCAGACACCAAACT-3' |
| II5'-GSP2 | 5'-CGAATTAGGATTCCAGTTGTT-3' |
| II5'-GSP3 | 5'-CGACCCCAATCAAGGCT-3' |

*CmSBE I* was amplified using SBEI-F and SBE I-R and *CmSBE II* was amplified using SBEII-F and SBE II-R. The gene-specific primer (GSP) used in 3’ RACE were I3'-GSP and II3'-GSP, respectively. Both *CmSBE I* and *CmSBE II* gene had 3 gene-specific primers for 5’ RACE. I5'-GSP1, I5'-GSP2 and I5'-GSP3 for *CmSBE I* gene, and II5'-GSP1, II5'-GSP2 and II5'-GSP3 for *CmSBE II* gene.
