# Supplementary material for: Identification and expression analysis of starch branching enzymes involved in starch synthesis during the development of chestnut (Castanea mollissima Blume) cotyledons
Source: PLoS One. 2017 May 23;12(5):e0177792. doi: 10.1371/journal.pone.0177792 (PMC5441625; doi:10.1371/journal.pone.0177792)
Supplement: S2 Table — (DOCX) [file pone.0177792.s003.docx]

**S2 Table. List of primers sequences used in qRT-PCR of the heat map.**

|  | forward primer | reverse primer |
| --- | --- | --- |
| *CmAGP* | 5’ - CCTCAATCGCCACATTTCAC–3’ | 5’ - AACCACTTCATTCCTGCTTCC –3’ |
| *CmSSS I* | 5’ - GCTCCATTAGTGCTTCCATTG–3’ | 5’ - GCTTGCGTGCCAGTCATT–3’ |
| *CmSSS II* | 5’ - CTTGGAGATGTTGCTGGTGCTT–3’ | 5’ - CTTCCGAACTCCTGTATCTTGG–3’ |
| *CmSSS III* | 5’ - GGTGGTGTGCTGATGTTGTT–3’ | 5’ - CTGGCGGCGGTTGTTATC–3’ |
| *CmSSS IV* | 5’ - ACAGTGGACATAATGCTTCCTTTC–3’ | 5’ - TGAGATGCCTGAAACTAACCC–3’ |
| *CmGBSS I* | 5’ - GCTATGAGAAGCCAGTGAAAG–3’ | 5’ - ACCAGTGATGCCAGTCTTAC–3’ |
| *CmGBSS II* | 5’ - CTTGGAGATGTTGCTGGTGCTT–3’ | 5’ - CTTCCGAACTCCTGTATCTTGG–3’ |
| *CmPUL* | 5’ - TCGTGATTCATACAACTCTG–3’, | 5’- ACCATCTTCAATGCTCATC–3’ |
| *CmSBE I* | 5’-CACAAGAATCATACTTCCACAC-3’ | 5’-CATCAGTTGCCTCACTAAAATAC-3’ |
| *CmSBE II* | 5’ - GCTAGGTCTGCTTGTTCTCAT–3’ | 5’- ATCTACATCAGTTGCCAATCCA–3’ |
| Actin | 5’– TTGACTATGAGCAGGAACTT-3’ | 5’-TTGTAGGTGGTCTCGTGAAT-3’ |
