# Supplementary material for: Identification and expression analysis of starch branching enzymes involved in starch synthesis during the development of chestnut (Castanea mollissima Blume) cotyledons
Source: PLoS One. 2017 May 23;12(5):e0177792. doi: 10.1371/journal.pone.0177792 (PMC5441625; doi:10.1371/journal.pone.0177792)
Supplement: S3 Table — ** P < 0.01, *p < 0.05; Past tem = pasting temperature, Area = average area of starch content, 30 μm2 = the proportion of starch granule ≤ 30 μm2, 60 μm2 = the proportion of starch granule between 30 μm2 and 60 μm2; 90 μm2 = the proportion of starch granule between 60 μm2 and 90 μm2; 220 μm2 = the proportion of starch granule between 90 μm2 and 220 μm2. (DOCX) [file pone.0177792.s004.docx]

**S3 Table. Correlation analysis between the gene expressions and some index of starch properties.**

| Gene expression | Past tem | Area | 30 μm^2^ | 60 μm^2^ | 90 μm^2^ | 220 μm^2^ |
| --- | --- | --- | --- | --- | --- | --- |
| *GBSS I* | -0.091 | -0.103 | -0.023 | 0.084 | 0.061 | -0.084 |
| *SSS I* | -0.311 | 0.328 | -0.181 | -0.085 | 0.282 | 0.258 |
| *SSS III* | -0.330 | 0.350 | -0.205 | -0.045 | 0.296 | 0.271 |
| *SBE I* | -0.809^*^ | 0.843^**^ | -0.800^*^ | 0.441 | 0.818^*^ | 0.848^**^ |
| *SBE II* | -0.872^**^ | 0.811^*^ | -0.829^*^ | 0.553 | 0.904^**^ | 0.724^*^ |

** P < 0.01, *p < 0.05; Past tem = pasting temperature, Area = average area of starch content, 30 μm^2^ = the proportion of starch granule ≤ 30 μm^2^, 60 μm^2^ = the proportion of starch granule between 30 μm^2^ and 60 μm^2^; 90 μm^2^ = the proportion of starch granule between 60 μm^2^ and 90 μm^2^; 220 μm^2^ = the proportion of starch granule between 90 μm^2^ and 220 μm^2^.
