# Supplementary material for: Identification and expression analysis of starch branching enzymes involved in starch synthesis during the development of chestnut (Castanea mollissima Blume) cotyledons
Source: PLoS One. 2017 May 23;12(5):e0177792. doi: 10.1371/journal.pone.0177792 (PMC5441625; doi:10.1371/journal.pone.0177792)
Supplement: S4 Table — Name, name of chestnut protein matched to the peptide; MS/MS peptide sequence, detected peptide sequence of chestnut by MS/MS; Pep_score, the score of the peptide; Pep_ expect, credibility evalution of the peptide; Mr_exp, expected relative molecular weight of the peptide; Mr_calc, calculated relative molecular weight of the peptide; Identified protein, identified protein using the MASCOT research engine and Swissprot2015 (Taxonomy: Viridiplantae [Green Plants] [35,297 sequences]) Species, the species of the identified protein; NCBI Acc, accession number from NCBI database of identified protein; Protein description, matched protein description; Mascot score, score obtained from MASCOT for each match; SC, amino acid sequence coverage for the identified proteins; Protein coverage was calculated on the basis of the amino acids (aa) identified and matched to the total number of aa in the protein sequence. (DOCX) [file pone.0177792.s005.docx]

**S4Table. List of peptides identified in SDS-PAGE by LC-MS/MS.**

| Name | MS/MS peptide sequence | Pep_ score | Pep_ expect | Mr_exp | Mr_calc | Identified proteins | Species | NCBI Acc. | Protein description | Mascot score | SC (%) |
| --- | --- | --- | --- | --- | --- | --- | --- | --- | --- | --- | --- |
| CmSBE I | FLLSNLR | 48.64 | 0.00012 | 861.51 | 861.51 | SBE 1 | *Solanum tuberosum* | P30924 | OS=Solanum tuberosum GN=SBE1 PE=2 SV=2 | 937.0 | 11% |
| CmSBE I | AMNLLDDK | 21.06 | 0.21 | 934.45 | 934.44 | SBE II | *Pisum sativum* | Q41059 | chloroplastic/amyloplastic (Fragment) OS=Pisum sativum GN=SBEIIPE=1 SV=1 | 858.4 | 11% |
| CmSBE I | IPAWIK | 28.83 | 0.0056 | 726.44 | 726.44 | SBE II | *Pisum sativum* | Q41059 | chloroplastic/amyloplastic (Fragment) OS=Pisum sativum GN=SBEIIPE=1 SV=1 | 858.4 | 11% |
| CmSBE I | IYEAHVGMSSSEPR | 16.7 | 0.18 | 1561.72 | 1561.72 | SBE II | *Pisum sativum* | Q41059 | chloroplastic/amyloplastic (Fragment) OS=Pisum sativum GN=SBEIIPE=1 SV=1 | 858.4 | 11% |
| CmSBE I | LAMAIPDKWIDYLK | 71.25 | 5.80E-07 | 1675.90 | 1675.90 | SBE II | *Pisum sativum* | Q41059 | chloroplastic/amyloplastic (Fragment) OS=Pisum sativum GN=SBEIIPE=1 SV=1 | 858.4 | 11% |
| CmSBE I | YKFMNAFDR | 20.11 | 0.1 | 1206.55 | 1206.55 | SBE II | *Pisum sativum* | Q41059 | chloroplastic/amyloplastic (Fragment) OS=Pisum sativum GN=SBEIIPE=1 SV=1 | 858.4 | 11% |
| CmSBE II | LHMAIADK | 34.65 | 0.0032 | 897.48 | 897.47 | SBE 2.2 | *Arabidopsis thaliana* | Q9LZS3 | chloroplastic/amyloplastic OS=Arabidopsis thaliana GN=SBE2.2 PE=2 SV=1 | 438.0 | 13% |
| CmSBE II | EWAPGAK | 25.89 | 0.017 | 757.38 | 757.38 | SBE 2.1 | *Arabidopsis thaliana* | O23647 | chloroplastic/amyloplastic OS=Arabidopsis thaliana GN=SBE2.1 PE=2 SV=1 | 434.0 | 15% |
| CmSBE II | GYHWMWDSR | 24.23 | 0.0087 | 1236.52 | 1236.51 | SBE 2.1 | *Arabidopsis thaliana* | O23647 | chloroplastic/amyloplastic OS=Arabidopsis thaliana GN=SBE2.1 PE=2 SV=1 | 434.0 | 15% |
| CmSBE II | IRMDTPSGIK | 14.13 | 0.6 | 1132.59 | 1132.59 | SBE 2.1 | *Arabidopsis thaliana* | O23647 | chloroplastic/amyloplastic OS=Arabidopsis thaliana GN=SBE2.1 PE=2 SV=1 | 434.0 | 15% |
| CmSBE II | MDTPSGIKDSIPAWIK | 41.74 | 0.0015 | 1757.91 | 1757.90 | SBE 2.1 | *Arabidopsis thaliana* | O23647 | chloroplastic/amyloplastic OS=Arabidopsis thaliana GN=SBE2.1 PE=2 SV=1 | 434.0 | 15% |
| CmSBE II | RFDLGDAEYLR | 68.86 | 1.50E-06 | 1353.67 | 1353.67 | SBE 2.1 | *Arabidopsis thaliana* | O23647 | chloroplastic/amyloplastic OS=Arabidopsis thaliana GN=SBE2.1 PE=2 SV=1 | 434.0 | 15% |
| CmSBE II | TIAFWLMDK | 28.99 | 0.015 | 1123.58 | 1123.57 | SBE 2.1 | *Arabidopsis thaliana* | O23647 | chloroplastic/amyloplastic OS=Arabidopsis thaliana GN=SBE2.1 PE=2 SV=1 | 434.0 | 15% |
| CmSBE II | VIVFER | 26.73 | 0.022 | 761.45 | 761.44 | SBE 2.1 | *Arabidopsis thaliana* | O23647 | chloroplastic/amyloplastic OS=Arabidopsis thaliana GN=SBE2.1 PE=2 SV=1 | 434.0 | 15% |
| CmSBE II | WWLEEYKFDGFR | 76.47 | 2.40E-07 | 1674.79 | 1674.78 | SBE 2.1 | *Arabidopsis thaliana* | O23647 | chloroplastic/amyloplastic OS=Arabidopsis thaliana GN=SBE2.1 PE=2 SV=1 | 434.0 | 15% |
| CmSBE II | WWLEEYK | 14.22 | 0.5 | 1052.50 | 1052.50 | SBE 2.1 | *Arabidopsis thaliana* | O23647 | chloroplastic/amyloplastic OS=Arabidopsis thaliana GN=SBE2.1 PE=2 SV=1 | 434.0 | 15% |
| CmSBE II | YLLSNAR | 13.79 | 0.46 | 835.46 | 835.46 | SBE 2.1 | *Arabidopsis thaliana* | O23647 | chloroplastic/amyloplastic OS=Arabidopsis thaliana GN=SBE2.1 PE=2 SV=1 | 434.0 | 15% |
| CmSBE II | INTYANFRDDVLPR | 41.29 | 0.001 | 1692.86 | 1692.86 | SBE I | *Pisum sativum* | Q41058 | chloroplastic/amyloplastic OS=Pisum sativum GN=SBEIPE=1 SV=1 | 313.0 | 9% |
| CmSBE II | MGDIVHTLTNR | 57.78 | 2.50E-05 | 1255.64 | 1255.63 | SBE I | *Pisum sativum* | Q41058 | chloroplastic/amyloplastic OS=Pisum sativum GN=SBEIPE=1 SV=1 | 313.0 | 9% |
| CmSBE II | WIELLKK | 0.05 | 5.3 | 928.58 | 928.57 | SBE I | *Pisum sativum* | Q41058 | chloroplastic/amyloplastic OS=Pisum sativum GN=SBEIPE=1 SV=1 | 313.0 | 9% |
| CmSBE II | YHGMQEFDR | 19.61 | 0.037 | 1181.50 | 1181.49 | SBE I | *Pisum sativum* | Q41058 | chloroplastic/amyloplastic OS=Pisum sativum GN=SBEIPE=1 SV=1 | 313.0 | 9% |
| CmSBE I | AHSLGLR | 17.85 | 0.11 | 752.43 | 752.43 | SBE 1 | *Oryza sativa* | Q01401 | chloroplastic/amyloplastic OS=Oryza sativa subsp. japonica GN=SBE1 PE=1 SV=2 | 283.0 | 9% |
| CmSBE I | LFNYANWEVLR | 67.05 | 2.40E-06 | 1423.73 | 1423.72 | SBE 1 | *Oryza sativa* | Q01401 | chloroplastic/amyloplastic OS=Oryza sativa subsp. japonica GN=SBE1 PE=1 SV=2 | 283.0 | 9% |
| CmSBE I | SGTPEDLK | 51.52 | 8.40E-05 | 845.41 | 845.41 | SBE 1 | *Oryza sativa* | Q01401 | chloroplastic/amyloplastic OS=Oryza sativa subsp. japonica GN=SBE1 PE=1 SV=2 | 283.0 | 9% |
| CmSBE I | VGCDLPGK | 48.7 | 0.00013 | 844.41 | 844.41 | SBE 1 | *Oryza sativa* | Q01401 | chloroplastic/amyloplastic OS=Oryza sativa subsp. japonica GN=SBE1 PE=1 SV=2 | 283.0 | 9% |
| CmSBE I | VIVFER | 26.73 | 0.022 | 761.45 | 761.44 | SBE 1 | *Oryza sativa* | Q01401 | chloroplastic/amyloplastic OS=Oryza sativa subsp. japonica GN=SBE1 PE=1 SV=2 | 283.0 | 9% |

Name, name of chestnut protein matched to the peptide; MS/MS peptide sequence, detected peptide sequence of chestnut by MS/MS; Pep_score, the score of the peptide; Pep_ expect, credibility evalution of the peptide; Mr_exp, expected relative molecular weight of the peptide; Mr_calc, calculated relative molecular weight of the peptide; Identified protein, identified protein were obtained with MASCOT research engine using swissprot2015(Taxonomy: Viridiplantae (Green Plants) (35,297 sequences))Species, the species of the identified protein; NCBI Acc, accession number from NCBI database of identified protein; Protein description, matched protein description; Mascot score, score obtained from Mascot for each match; SC, amino acid sequence coverage for the identified proteins; Protein coverage is calculated on the basis of the amino acids (aa) identified and matched to the total number of aa in the protein sequence.
